# Introduction of Soft X-Ray Spectromicroscopy as an Advanced Technique for Plant Biopolymers Research

**DOI:** 10.1371/journal.pone.0122959

**Published:** 2015-03-26

**Authors:** Chithra Karunakaran, Colleen R. Christensen, Cedric Gaillard, Rachid Lahlali, Lisa M. Blair, Vijayan Perumal, Shea S. Miller, Adam P. Hitchcock

**Affiliations:** 1 Canadian Light Source Inc., 44 Innovation Boulevard, Saskatoon, Saskatchewan, Canada; 2 Industrial Research Assistance Program—National Research Council Canada, 110 Gymnasium Place, Saskatoon, Saskatchewan, Canada; 3 INRA—Biopolymers, Interactions, Assemblies Unit (BIA), Nantes, France; 4 Canadian Food Inspection Agency, 116 Veterinary Road, Saskatoon, Saskatchewan, Canada; 5 Agriculture and Agri-Food Canada, *Eastern Cereal* and *Oilseed Research Centre*, *Ottawa*, *Ontario*, *Canada*; 6 Brockhouse Institute for Materials Research, McMaster University, 1280 Main Street West, Hamilton, Ontario, Canada; SPECS Surface Nano Analysis GmbH, GERMANY

## Abstract

Soft X-ray absorption spectroscopy coupled with nano-scale microscopy has been widely used in material science, environmental science, and physical sciences. In this work, the advantages of soft X-ray absorption spectromicroscopy for plant biopolymer research were demonstrated by determining the chemical sensitivity of the technique to identify common plant biopolymers and to map the distributions of biopolymers in plant samples. The chemical sensitivity of soft X-ray spectroscopy to study biopolymers was determined by recording the spectra of common plant biopolymers using soft X-ray and Fourier Transform mid Infrared (FT-IR) spectroscopy techniques. The soft X-ray spectra of lignin, cellulose, and polygalacturonic acid have distinct spectral features. However, there were no distinct differences between cellulose and hemicellulose spectra. Mid infrared spectra of all biopolymers were unique and there were differences between the spectra of water soluble and insoluble xylans. The advantage of nano-scale spatial resolution exploited using soft X-ray spectromicroscopy for plant biopolymer research was demonstrated by mapping plant cell wall biopolymers in a lentil stem section and compared with the FT-IR spectromicroscopy data from the same sample. The soft X-ray spectromicroscopy enables mapping of biopolymers at the sub-cellular (~30 nm) resolution whereas, the limited spatial resolution in the micron scale range in the FT-IR spectromicroscopy made it difficult to identify the localized distribution of biopolymers. The advantages and limitations of soft X-ray and FT-IR spectromicroscopy techniques for biopolymer research are also discussed.

## Introduction

A good understanding of the structural organization, chemical composition, and correlation between structure and composition of biopolymers in plants and plant products is essential to continually improve quality by plant breeding, to preserve quality through processing and storage, and to extend efficient utilization through new product development. Electron microscopy (EM), analytical chemistry, and histochemical methods are extensively used to characterize biopolymers in plant products [1–3]. These methods are limited by the lack of sensitivity and information loss on the spatial localization and distribution of chemical components. Fixation and staining protocols used in EM and histochemical analyses affect chemical characterization and quantitative information. Chemical extraction methods may alter the original compound and produce derivatives that interfere with the analysis [4]. Vibrational (Raman and infrared) and ultraviolet spectromicroscopy techniques have long been used as non-destructive methods for in-situ physicochemical characterization of biopolymers [5,6]. Characterization of seeds (lentils, pea, wheat, corn, oats, rye, onion), fibres (flax, hemp), grass (rye grass), and plant residues (wheat straw, poplar wood) by either laboratory- or synchrotron-based Fourier Transform mid Infrared (FT-IR), Raman, and ultraviolet spectromicroscopy methods have been reported [1,3,6–18]. Although lots of work have been reported on biopolymer characterization, an in-depth understanding on the localization of biopolymers, their interactions and contribution to diverse functions is necessary.

The wavelength of light provides a limit to the spatial resolution and chemical information obtained from a sample. The wavelength of IR light is in the micrometer range (4000 cm^-1^–200 cm^-1^, or 2.5 μm–50 μm) and limits the spatial resolution to much less than that obtained using a visible light microscope (300–500 nm). Soft X-rays on the other hand have shorter wavelengths in the nanometre range (100 eV–2500 eV, or 12 nm – 0.5 nm). Therefore, soft X-rays have the potential to provide much high spatial resolution and thus can characterize samples at the sub-cellular (nanometer scale) level. In this study, soft X-ray spectromicroscopy using Scanning Transmission X-ray Microscope (STXM) is shown to be a powerful technique that can be used to characterize plant samples at a high spatial resolution and similar chemical sensitivity compared to mid infrared spectromicroscopy. Recent advancement in the fabrication of zone plates which focus the X-ray beam has made it possible to achieve a spatial resolution of up to ~ 10 nm using STXM [19].

Soft X-ray spectromicroscopy is a synchrotron based technique for elemental identification, elemental speciation, and spatial mapping of heterogeneous materials [20]. When monochromatic X-ray beam is incident on a sample, it is absorbed and excites core electrons from a specific atom in a molecule to unoccupied molecular orbitals giving rise to near edge X-ray absorption spectra (XAS) around the elemental absorption edges [21]. The XAS structures are closely related to chemical bonding and can be used to determine and quantify the presence of elements or compounds, similar to mid infrared (IR) spectroscopy [22–25]. Using STXM, XAS of samples can be collected at each spot on thin sections of samples by raster scanning the samples. The STXM has been extensively used for characterization of polymer materials [26,27]; environmental samples [28–32]; and biomaterials for medical applications [33–35]. Only a very few work has been reported on the use of STXM for plant biopolymer research such as characterization of plant fossil and xylem lignification [28,36–39] and DNA distribution in bean chromosomes [40,41].

Physicochemical characterization of plant biopolymers at the cellular (micron scale) and sub-cellular level helps to develop desired products as well as to maximize the benefits. Some examples include: studying changes in cell composition and structure during seed development [42–44]; correlation between plant cell wall composition and its susceptibility to diseases or final product quality [1,45–47]; determining stem or wood composition and using plant breeding programs to increase or reduce components like lignin [7,15]; characterization of fibres to optimize processing procedures and to improve the quality of biocomposites [13,48]; and understanding of bio-wastes to maximize by-product development like extraction of cellulose and hemicellulose [11]. Therefore, the objectives of this study were to: 1) compare the sensitivity of soft X-ray absorption spectroscopy (XAS) with that of FT-IR spectroscopy in identification of common plant biopolymers; 2) determine the advantages of soft X-ray spectromicroscopy for biopolymer localization and distribution in plant samples; and 3) compare the relative merits of soft X-ray and FT-IR spectromicroscopy techniques for biopolymer research.

## Materials and Methods

### Biopolymer References

To achieve objective 1, spectra of the following biopolymer references were recorded using the soft X-ray XAS and FT-IR spectroscopy methods: Lignin (hydrolytic), cellulose, hemicelluloses (xylan from oat spelts; hot water insoluble xylan; hot water soluble xylan; wheat arabinoxylan), pectin (from orange, polygalacturonic acid-PGA) and β-glucan (from oats). The reference compounds were purchased either from Sigma-Aldrich Canada Ltd or Megazyme International Ireland Ltd.

### Lentil Stem Section

For objective 2, the localization and distribution of biopolymers were mapped at the cellular (FT-IR) and sub-cellular level (soft X-ray) on lentil stem sections. Stem sections (from the 3^rd^ or 4^th^ nodes) excised from lentil plant (*Lens culinaris;* variety VIR 421) were used to prepare the samples.

### Samples for Soft X-ray Spectromicroscopy

The soft X-ray absorption spectra of biopolymer references were collected using STXM in the transmission mode. In STXM, for ideal transmission through the sample (about 30–60%), samples have to be thin (for carbon XAS, samples have to ~90–200 nm thick if the density of the material is about 1 g/cm^3^) so that soft X-rays can penetrate the sample. Therefore, each reference compound was either suspended or dissolved in water and about ~ 2 μl droplet was deposited on the flat side of a Silicon Nitride (Si_3_Ni_4_) window (1 mm x 1 mm pane, 75 nm thick, Norcada Ltd) and allowed to dry in air.

For soft X-ray spectromicroscopy, lentil stem sections (~ 1–2 mm^3^) were dehydrated in a graded ethanol series from 25 to 100% in four steps of 20–30 min at each concentration. Dehydrated sections were then embedded in an amine epoxy resin. The resin with the sample was polymerized at 60°C for 24 h or until the resin was completely polymerized. The embedded sections were cut at room temperature to a thickness of ~90 nm using an Ultramicrotome. The cut sections were then deposited on uncoated copper grids commonly used for transmission electron microscopy.

### Samples for FT-IR Spectromicroscopy

The FT-IR spectroscopy of biopolymer references were collected using an IR photoacoustic cell. The three common modes of IR spectroscopy are attenuated total reflectance (ATR), diffuse reflectance, and photoacoustic spectroscopy (PAS). Of these methods, PAS requires no or minimum sample preparation and the PAS is similar to ATR and transmission spectra [18,49]. Therefore, PAS was used in this study. The PAS determines the absorption in the infrared region by measuring the changes in the thermal expansion of the gas surrounding the sample using a microphone.

For infrared spectromicroscopy in the transmission mode, lentil stem sections of about 1 cm long were frozen at -20°C for about 16 h. Cross sections of ~ 8 μm thickness were cut using the cyro-microtome (Leica CM3050 S—Cryostats, Leica Biosystems). The sections were deposited on 3 mm thick barium fluoride (BaF_2_) slides that are suitable for IR data collection in the transmission mode.

### Soft X-ray Spectromicroscopy Data Collection

The XAS data of the biopolymer references and lentil stem sections were recorded using the interferometrically controlled STXMs at the Canadian Light Source (CLS)—soft X-ray spectromicroscopy beamline (beamline 10ID-1) and Advanced Light Source (ALS)—Polymer STXM (beamline 5.3.2). Both the CLS and ALS beamlines are optimized for spectromicroscopy at the C, N, and O 1s edges suitable for biopolymers research and provide monochromatic soft X-ray beam in the energy range of 130–2700 eV and 250–700 eV, respectively. The monochromatic X-ray beam was focused to a spot size of ~40 nm by the 35 nm outer-zone diameter zone plate on the sample. The transmitted X-ray intensity through the sample was recorded by a detector (a thin phosphor coating converts X-rays to visible light and a single photon counting unit gives the intensity counts). The beamline slit sizes were selected so that the spectral resolving power was ~3000 at the C 1s and O 1s regions. A 200 nm thick Titanium (Ti) filter was used at the 10ID-1 beamline to reduce the contribution from higher order light in the incoming beam at the C 1s region and a gas cell filled with 7 Torr N_2_ over a 1 m pathlength was used for second order reduction at the ALS 5.3.2 beamline. The energy scale of both beamlines was checked by recording the gas spectra of CO_2_ and all data sets were corrected for the correct energy scale [50].

After the sample was mounted into the microscope, the chamber was first evacuated to the lowest vacuum achievable (~ 100 m Torr). The microscopic chamber was then filled with dry helium (~1/3 of atmospheric pressure) to provide cooling for motors inside the microscope. The absorption spectra of the dried samples were recorded at the C 1s (280–320 eV) and O 1s (525–560 eV) regions. The samples were checked for radiation damage after recording the data, as radiation damage may alter the functional groups of biopolymers [38,51]. The intensity of the incoming beam recorded through the Si_3_Ni_4_ window where there was no sample was used to normalize the transmitted intensity through the sample.

In STXM, XAS data can be collected in three different modes: point spectra at a single location, line scan along a line in a sample, or a stack to record images over a sequence of photon energies. Spectra of the reference compounds were recorded using a defocused point scan (~800 ms dwell time per point or pixel), or a line scan (~8 to 10 ms dwell time per line) or a stack (an image sequence at 1 ms dwell time per pixel) at an energy step size of 0.07 eV around the C 1s absorption edge of different functional groups (284.5 to 293 eV). The lentil stem section was analyzed by recording an image sequence in the C 1s region (280 to 300 eV) around cell wall and cell wall junctions. A spatial sampling of 100 nm, energy resolution of 0.2 eV, an energy range from 284.2 to 292 eV, and a dwell time of 1 ms took about ~35 min to record a stack from a region of 14 μm×16 μm on the lentil stem section.

### FT-IR Spectromicroscopy Data Collection

The IR spectra of biopolymer references and lentil stem sections were collected at the CLS Mid-IR beamline (beamline 01B1-1, energy range: 4000–400 cm^-1^). The beamline has a MTEC Model 300 photoacoustic cell (MTEC Photoacoustics Inc., Ames, IA) for spectroscopy of bulk samples and a Bruker Optics IFS66vs FT-IR (Bruker Optics Inc., Billerica, MA) confocal microscope equipped with a potassium bromide beam splitter, motorized mapping stage, and cryo-cooled mercury cadmium telluride detector for spectromicroscopy of thin samples. The microscope is configured to use synchrotron light as well as the built-in Globar source.

The FT-IR spectra of reference compounds were recorded using the PAS system using the Globar source. The reference compounds were placed in the sample cup and the sample chamber was purged with dry helium to remove water vapour and CO_2_. The spectrum for each sample was recorded by averaging 32 interferograms collected from wavelengths 4000 to 800 cm^-1^ at a resolution of 4 cm^-1^. The spectrum of carbon black powder (average of 4 trials with 32 scans per trial) was also recorded to normalize the sample spectrum.

Although infrared spectroscopy and microscopy are available based on Globar source, the synchrotron source have better spatial resolution (3–10 μm) and signal-to-noise ratio (100–1000 times) [52]. Therefore, the FT-IR spectromicroscopy data of lentil stem sections were recorded in the transmission mode using synchrotron light at a spectral resolution of 8 cm^-1^ in the 4000–800 cm^-1^ energy range. A visible light microscope equipped with a CCD camera connected to the FT-IR microscope was used to identify regions of interest. The sample chamber was purged with dry helium to minimize IR absorption by water vapour and CO_2_. A spatial resolution of 5 μm × 5 μm was used for microscopy. A total of 128 scans were recorded at each spot on the sample and the average of the scans was normalized using a background spectrum (average of 512 scans) obtained from an area where there was no sample. The total time for collecting data on an area of 225 μm × 45 μm was about 8 h.

### Soft X-ray Data Analysis

All STXM data were analyzed using aXis2000 program (http://unicorn.mcmaster.ca/aXis2000.html). The method is described in detail elsewhere [25,53]. The XAS spectra of biopolymer references were first converted to optical densities (OD) using a spectrum recorded where there was no sample. The theoretical absorption or optical density of a 1 nm thick biopolymer reference was computed assuming appropriate elemental composition and density. For example, densities of 1.5 and 1.3 g/cm^3^ were assumed for cellulose and lignin, respectively. The experimental biopolymer reference spectra were then normalized to absorption by 1 nm of corresponding compound. A linear background was subtracted from each experimental reference spectrum to match the pre-edge and post-edge to the calculated theoretical absorption spectrum.

The stack data of lentil stem section after aligned and converted into OD was transformed into component maps. The images in the stack data were aligned using the cross correlation method using an image (287.1 eV) which had the highest contrast. The aligned stack was then converted into OD using a spectra extracted from an empty region within the stack. The normalized and background corrected experimental reference spectra of lignin and cellulose were used to determine the spatial and quantitative distribution of biopolymers in the lentil stem section. A singular value decomposition method (SVD) using the spectra of reference compounds was used to map and to determine the quantitative distribution of biopolymers [20,25,53].

### Infrared Data Analysis

OPUS 4.2 (Bruker Optics Inc., Billerica, MA) software was used to record and analyze the PAS and FT-IR spectromicroscopy data. The PAS biopolymer reference spectra were first normalized using the carbon black spectra and then smoothed by an average filter (3 to 9 points). Origin (version 7.5) was used for plotting all the spectral data.

The FT-IR spectromicroscopy data was first normalized using the spectra recorded through the BaF_2_ disc where there was no sample. The baseline of the lentil stem section spectromicroscopy data was then corrected using the rubber-band correction (64 points). The second derivative of the FT-IR absorbance spectrum from the biopolymer references was used to identify integration band ranges for lignin and cellulose. For example, lignin and cellulose distribution on the lentil stem section was determined by integrating the spectral ranges of 1524–1502 cm^-1^ and 1442–1417 cm^-1^, respectively.

## Results and Discussion

### Spectroscopy of Biopolymer References

#### Soft X-ray Spectroscopy

**Fig 1:**
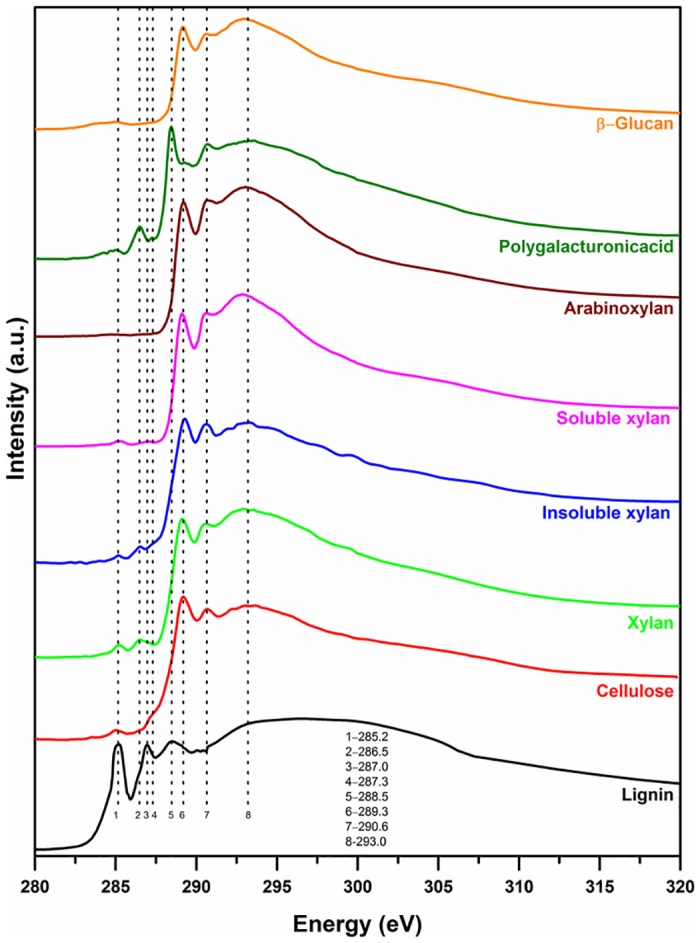
Carbon 1s soft X-ray absorption spectra of lignin, cellulose, xylan, arabinoxylan, polygalacturonic acid, and β-glucan.

For water insoluble compounds like lignin and cellulose, thin regions of prepared samples were chosen (using Leica refraction color chart from the optical microscope images or using the optical density from the STXM images) to optimize transmission through the sample at the C 1s and O 1s absorption edges. The XAS spectra of biopolymers at the C 1s are shown in Fig 1. The three prominent 1s→π* transition peaks of lignin at 285.2, 287.0, and 288.5 eV are representative of the C and H substituted aromatic carbon, O substituted aromatic carbon, and carboxylic group [28]. The peaks at 285.2 and 287.0 eV are unique to lignin and thus lignin can be easily differentiated from other biopolymers. The differences in peak intensities at 285.2 and 287.0 eV are based on the relative amounts of *p*-coumaryl, coniferyl, or sinapyl monomers present in lignin from different plants [38]. Cellulose, water insoluble xylan, and water soluble xylan had strong 1s→π* transition peaks at 289.3 and 290.7 eV that are associated aliphatic C-OH and C-H bonds, respectively. Small shoulders were present in the three samples at 285.2 and 286.5 that are associated with aryl C-H and phenol-C, respectively. Compared to xylans, the 286.5 eV peak was very weak for cellulose. The transition peaks of the cellulose spectrum are in agreement with earlier work [38]. The relative peak intensities at 285.2 and 286.5 eV for water soluble and insoluble xylans were different. However, the differences are very subtle and may be difficult to be used as a marker for differentiation. Arabinoxylan did not have the 285 and 286.5 eV shoulder peaks but its other transition peaks were similar to those found in the xylans and cellulose. The PGA had a distinct spectrum with a broad transition peak at 285.2 eV, an intense peak at 286.5 eV, and a distinct peak at 288.5 eV (carboxyl group) and hence it can be easily distinguished from other biopolymers. A small shoulder peak was also present at 287.3 eV in the PGA spectrum. β-glucan had a broad transition peak at 285.2 eV and a prominent transition peak at 289.3 eV. The 1s→σ* transition peak at 293.0 eV for the aromatic and aliphatic carbon are very broad and were present in the spectra of all compounds studied.

**Fig 2:**
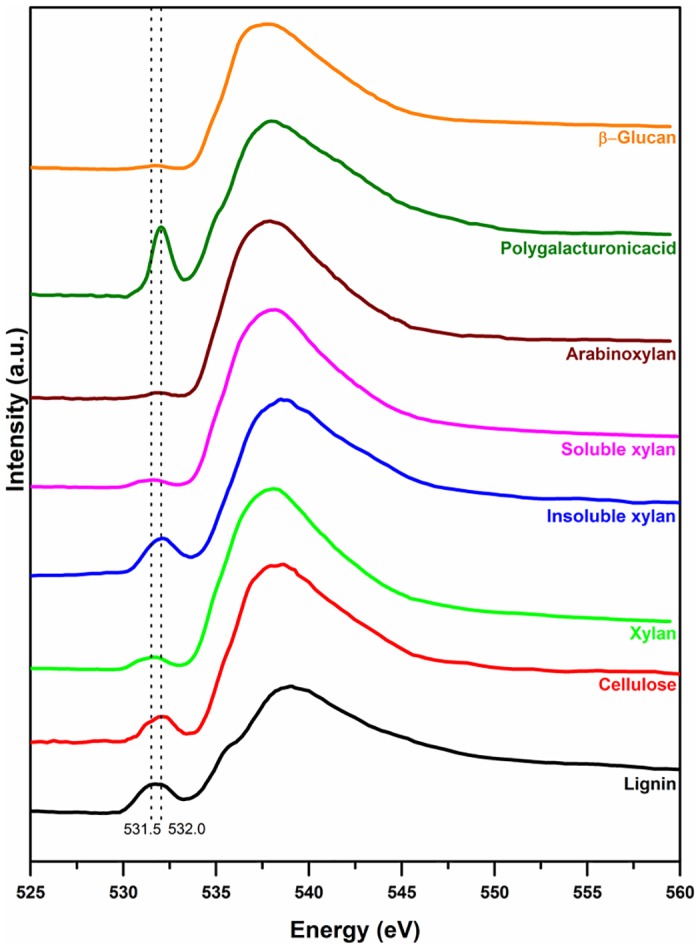
Oxygen 1s soft X-ray absorption spectra of lignin, cellulose, xylan, arabinoxylan, polygalacturonic acid, and β-glucan.

The O 1s XAS spectra of all biopolymers were similar except the PGA spectrum (Fig 2). Therefore, O 1s spectra by themselves cannot be used to identify different plant biopolymers. The 531.5 and 532.0 eV peaks can be assigned to the 1s→π* peak of C = O and the broad peak around 538 and 539 eV are due to the σ*-resonances [54].

#### FT-IR Spectroscopy

**Fig 3:**
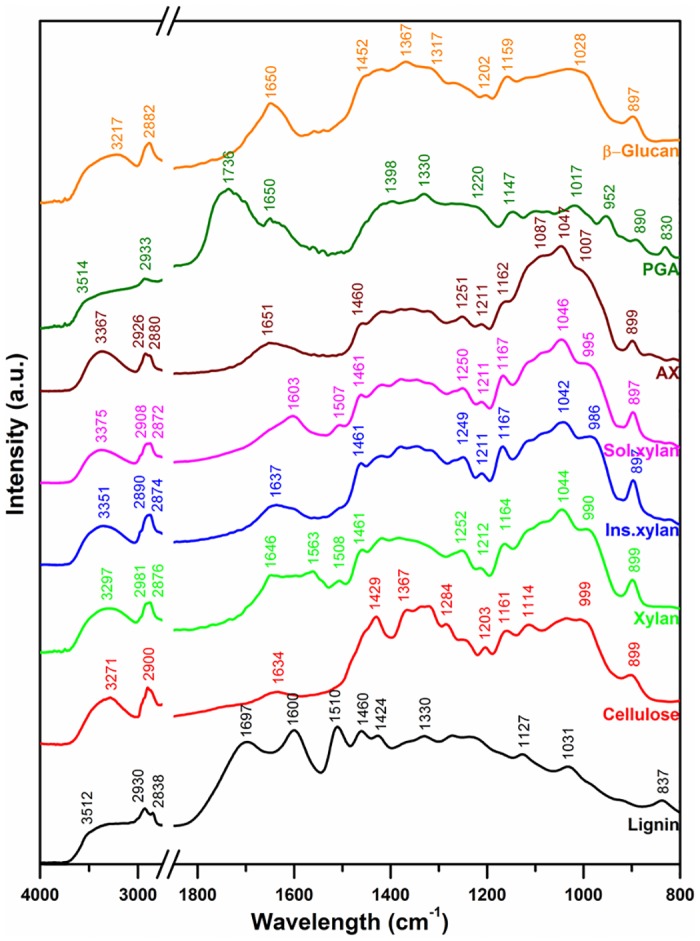
FT-IR photo acoustic spectra of lignin, cellulose, xylan, arabinoxylan, polygalacturonic acid, and β-glucan.

Fig 3 shows the IR spectra of lignin, cellulose, and hemicellulose, and PGA. The broad peak at 3520–3200 cm^-1^ of all compounds represents OH stretching vibrations [55,56]. Aliphatic C-H vibrations occur near 3000 cm^-1^ and CH_2_ groups have doublets peaks around 2900 and 2800 cm^-1^ [55,56]. The vibrational peaks between 2935–2915 and 2850–2815 cm^-1^ are attributed to methylene C-H stretch and methoxy ether (O-CH_3_) groups, respectively. All biopolymers have several similar and some distinct vibrational peaks in the 1800–800 cm^-1^ region. Therefore, the vibrational peaks in this region are useful to finger print and identify different biopolymers.

The lignin spectrum had characteristic peaks at 1697, 1600, 1510, and 837 cm^-1^. Smaller peaks were present at 1460, 1424, 1330, 1127, and 1031 cm^-1^. The 1600 cm^-1^ peak is attributed to the C = C vibration [55]. The peaks at 2930 and 2838 cm^-1^ confirm the presence of methylene C-H stretch and methoxy ether (O-CH_3_) groups in lignin. Vibrational peaks for lignin are observed at 1595 and 1510 cm^-1^ in corn kernels [6]; at 1515 cm^-1^ in wheat seed [12], and at 1610, 1602, and 1502 cm^-1^ in soft and hard woods [57]. The 1424 and 1330 cm^-1^ peaks are associated with the vinyl C-H in-plane bend and methylene C-H bend. In general, the peak at 1510 cm^-1^ is considered characteristic for lignin.

The cellulose spectrum had three distinct vibrational peaks at 1634, 1429, and 899 cm^-1^. Small vibrational peaks or shoulders were present at 1367, 1284, 1203, 1161, 1114, and 999 cm^-1^. The vibration at 2900 cm^-1^ is attributed to the CH stretch and bands between 1460–1200 cm^-1^ are attributed to the CH_2_, CH, and OH deformations. The band at 899 cm^-1^ is attributed to the pyranose sugar (917±13 cm^-1^) and confirms the β-linkage (891±7 cm^-1^) present in cellulose whereas α-monomers have a band at 844±8 cm^-1^ [56]. The 1429 cm^-1^ vibrational peak of cellulose is dominant and is distinct compared to spectra of hemicellulose, PGA, and β-glucan.

Xylan had two prominent vibrational peaks at 1646 and 1563 cm^-1^ whereas the insoluble and soluble xylan had prominent peaks at 1637 cm^-1^ and 1603 cm^-1^, respectively. All other peaks of the xylan compounds at wavelengths from 1460 to 800 cm^-1^ were similar. The vibrational shoulder around the 990 cm^-1^ was slightly lower for insoluble xylan (986 cm^-1^) and higher for the soluble xylan (995 cm^-1^). Hettrich reported peaks of 1070, 980, 620, 450 cm^-1^ as characteristics to xylan from oat spelts [9]. Arabinoxylan had prominent vibrational peak at 1651 cm^-1^ some notable peaks at 1460, 1251, 1211, 1162, 1087, 1047, and 899 cm^-1^ which were all similar to xylan. The shoulder at 1007 cm^-1^ peak was shifted towards higher energy compared to the 990 cm^-1^ peaks of xylan compounds. Phillipe et al. [46] and Robert [10] assigned peaks of 1002**,** 984, 958, and 895 cm^-1^ to arabinoxylan from wheat and Cyran [1] assigned peak at 1045 cm^-1^ to arabinoxylan from rye. Xu et al. [11] assigned peaks of 1165 and 1044 cm^-1^ to arabinoxylan as well and they are all in agreement with the observed peaks in this study.

Polygalacturonic acid had a characteristic peak at 1736 cm^-1^, the vibrational peak is characteristic to ester groups (1750–1725 cm^-1^) [11]. Other characteristic peaks were at 1330 cm^-1^ (with two surrounding shoulders at 1397 and 1220 cm^-1^), 1147, 1017, 952, 890, and 830 cm^-1^. The peaks of PGA were different from all other biopolymers reported here and are in agreement to vibration peaks from onion pectin [14].

β-glucan had prominent vibrational peaks at 1650, 1367 (with two shoulders), 1202, 1159, and 897 cm^-1^. Small shoulders were present at 1452, 1317, 1202, and a broad band around 1028 cm^-1^. The 1650 cm^-1^ peak was present in xylan, and arabinoxylan. The 1367, 1202, and 1159 peaks were unique to β-glucan and thus β-glucan can be easily differentiated from other biopolymers. The peaks (1020, and 895 cm^-1^) reported as assigned to β-glucan from wheat in a previous study [10] are in agreement with this study. Wetzel et al. [58] reported 1420 cm^-1^ peak is representative for β-glucan but this peak is present in cellulose and hemicellulose as well.

### Plant Cell Spectromicroscopy

#### Soft X-ray Spectromicroscopy

**Fig 4:**
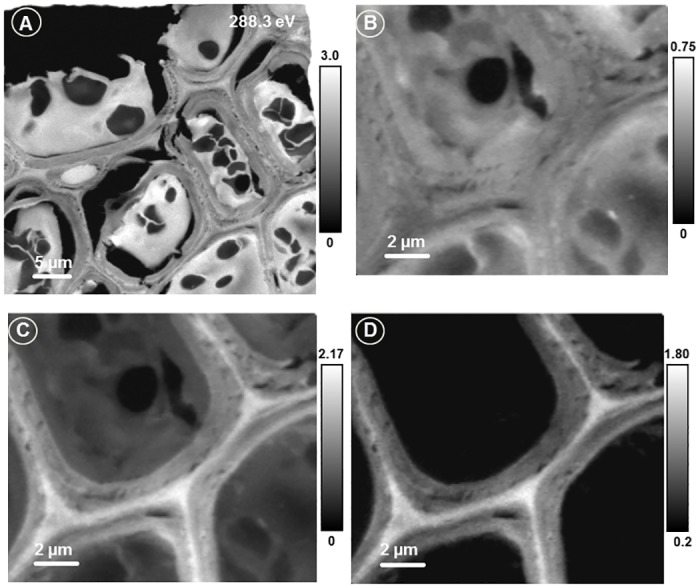
Soft X-ray microscopic images of lentil stem section. Image recorded at 288.3 eV (A) showing a large number of cells; Pre-edge (B: average from 282.0–283.0 eV), lignin absorption (C: average from 284.7–285.7 eV), and lignin distribution (D: C-B) images of lentil stem section. Scale bars indicate X-ray optical density.

The lentil stem cells were visible from the light microscopic image but did not show any internal structures or compositional differences between different cell components. The X-ray image of the sample (Fig 4A) recorded at 288.3 eV shows very clearly the individual cells, cell components, cell walls, and the middle lamella. The strong variation in absorption intensities in different regions is due to differences in absorption by different biopolymers. Fig 4 shows the advantages of the quick mapping method to determine the spatial distribution of any compound from a large area of a sample. The difference between two images (or the average of a few images around that energy region) recorded at the pre-edge (where non-carbon compounds or thickness effect of the sample shows up) and at the strong absorption peak of a compound reveals the spatial distribution of the compound. For example, Fig 4B and 4C shows X-ray image at the pre-edge (average from 282.0 to 283.0 eV) and around the characteristic absorption peak of lignin (average from 284.7 to 285.7 eV). The difference between the two images (Fig 4D) shows clearly the spatial distribution of lignin. Lignin was concentrated in the cell wall and middle lamella and there was variation in the distribution of lignin between the primary and secondary cell walls (Fig 4D).

**Fig 5:**
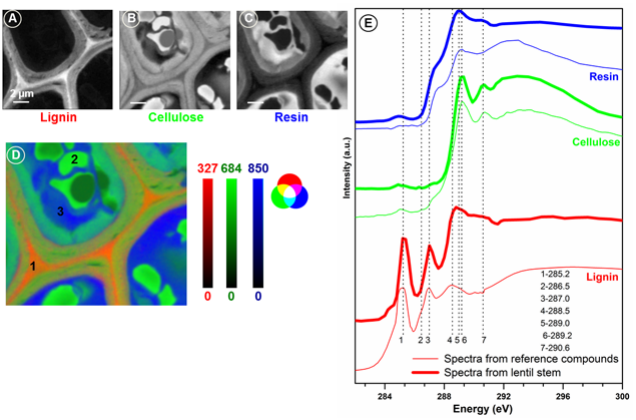
Mapping of lignin, cellulose, and resin in lentil stem section (A-C), and RGB composite image (D) showing the distribution of all three components in lentil stem section. Comparison of C 1s X-ray absorption spectra of reference compounds (E) with that of spectra extracted from three locations (D) on the lentil stem section. Scale bars indicate the thickness of compounds in nm.

Fig 5 shows the detailed spectromicroscopic data analysis of lentil stem section. The normalized reference spectra of lignin, cellulose, and resin were used to map and determine the quantity of biopolymers present (Fig 5A–5D, scales bars of each map show the thickness of individual components in nm). The spectra extracted from three marked regions (Fig 5D) of the sample were compared with the spectra of pure lignin, cellulose, and the amine epoxy resin (Fig 5E). Comparison of lignin spectra from the sample shows that the peak of 288.5 eV associated with lignin was shifted to ~ 290 eV as PGA is also present in high concentrations in the middle lamella of plant cells. The amine epoxy resin has a peak at 290 eV. It is likely that during polymerization of the sample and resin, the resin penetrated the sample. The resin bonded well with the compounds inside the cell except the middle lamella as the resin map does not show any absorption in the middle lamella. This was evident, as the spectra extracted from all locations inside the cell wall except the vacuoles or other structures resembled resin spectra (shown in blue in Fig 5D and 5E). It is interesting to note that the spectra from the vacuoles inside the cell wall resembled cellulose or hemicellulose spectra and were not contaminated by the resin spectra. Cell walls had higher concentrations of lignin and the concentration was highest in the middle lamella. In the primary and secondary cell walls, the occurrence of lignin and cellulose was clearly evident from the mixing of two spectral features represented by the colours of lignin and cellulose.

#### FT-IR Spectromicroscopy

**Fig 6:**
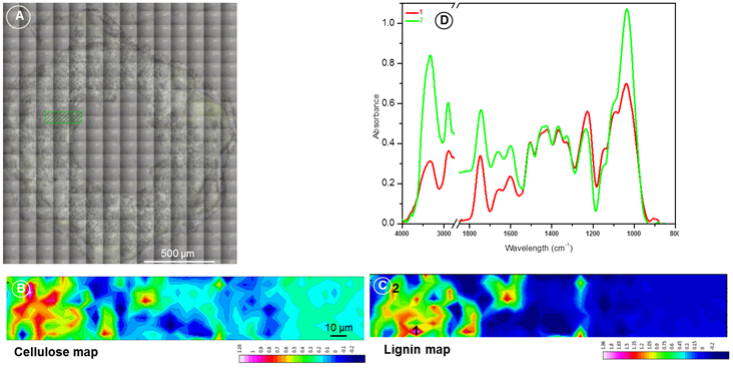
Visible (A) and FT-IR spectromicroscopy (B-D) of lentil stem section. Visible image of the entire lentil stem section (A) cut for FT-IR spectromicroscopy. The cellulose (B) and lignin (C) IR maps together with the spectra extracted (D) from two regions are shown.

The optical microscopic image recorded on the entire lentil stem section prepared for IR study is shown in Fig 6A. The stem section was approximately 844 μm in radius from the center and had a tissue thickness of about 540 μm from inside to outside (Fig 6A). The rectangular marked area (Fig 6A) represents the optical microscopic image of the scanned area, and extracted maps of lignin and cellulose from the stem section (Fig 6B and 6C). The lignin and cellulose maps showed that there was spatial correlation on the distribution of the two biopolymers. However, differentiation of individual cells and the cell components were difficult. The spectra (Fig 6D) extracted from two different marked regions (Fig 6C) on the stem section show that both lignin and cellulose were present in that locations.

#### Sensitivity of Soft X-ray Spectroscopy

Soft X-ray absorption spectra are elemental specific. For example, the carbon 1s absorption peak is about 285 eV and the oxygen 1s absorption peak is about 530.0 eV (http://xdb.lbl.gov/). In addition, the fine structures of the absorption spectra are representative of the local chemical bonds and functional groups [21]. The method has very good sensitivity to identify and characterize synthetic polymers [27,59]. It has been shown that the XAS of amino acid monomers, peptides (<50 amino acids), and small proteins with substantial different compositions are different [60]. However, the XAS of complex proteins or different proteins cannot be differentiated due to large number of similar monomers present which masks the differences [61–63]. Similar problem is encountered in the macromolecules of cellulose, hemicellulose, and β-glucan due to similar bonds and functional (C-C, C-H, C = O, C-OH, CH_2_) groups present in these biopolymers. The PGA due to the presence of carboxylic functional group is making it feasible to differentiate it from other biopolymers. The protein and starch C 1s spectra of plant derivatives have unique spectral features and can be easily differentiated from other biopolymers [64]. The reference spectra of biopolymers recorded in this study were collected at a spectral resolution of 0.07 eV and most soft X-ray beamline have a resolution of ~0.1 eV, therefore, increasing the spectral resolution may not help to differentiate cellulose, hemicellulose, and β-glucan in the soft X-ray regime.

Unlike soft X-ray spectroscopy, infrared spectroscopy is specific to molecular vibrations and is sensitive to identify the functional groups within a molecule. Therefore, the IR spectra of biopolymer references have unique spectral features.

#### Advantages of Soft X-ray Spectromicroscopy

The chemical absorption contrast combined with the nanometer resolution makes the soft X-ray spectromicroscopy an ideal tool for biopolymer characterization in-situ in samples. The use of soft X-ray spectromicroscopy for biopolymer characterization in seeds and flax-fiber composites was investigated recently [64–66]. The distribution of protein around starch granules on a pristine seed endosperm and the effect of chemical treatment on flax fiber samples were investigated using STXM for the first time.

**Fig 7:**
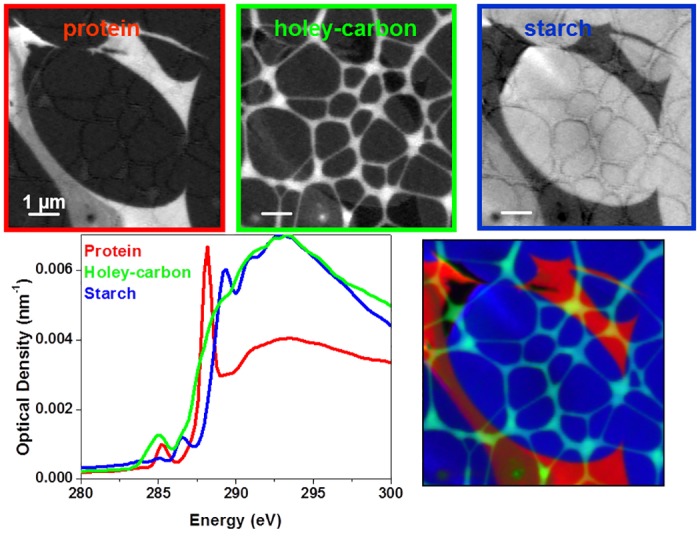
Compositional maps of starchy endosperm. Top row: component maps of protein, holey-carbon, and starch granules; bottom row: C 1s X-ray absorption spectra extracted from the components and the composite image showing all components in a single image.

**Fig 8:**
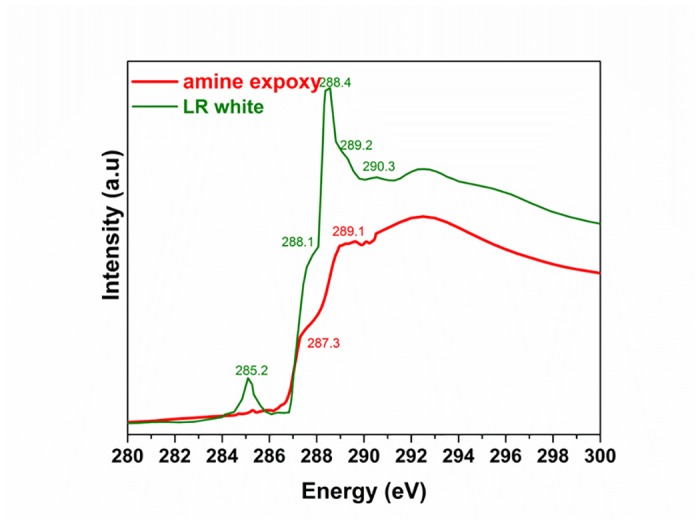
Carbon 1s soft X-ray absorption spectra of LR white and amine epoxy resin.

An application of soft X-ray spectromicroscopy in seeds is to characterize starch granules and biopolymers enclosing the granules [64]. The starch granules in seeds are of micrometer or sub-micrometer in size, soft X-ray spectromicroscopy is an ideal tool to investigate the starch granules and surrounding biopolymers. Further, in order to check an alternative sample preparation route avoiding the use of resins, ultrathin sections were cut by ultramicrotomy from native wheat grains without using any fixation and resin-embedding steps. This way was successfully applied to prepare 70 nm thick sections of the wheat starchy endosperm zone. The slices were placed on holey carbon coated copper grids. Fig 7 shows the results obtained from the starchy endosperm sample. Starch granules are enclosed by a layer of protein confirmed by the spectral signature differences. Staining techniques have been used to determine different components after observing differences in electron densities in different regions as observed using a scanning or transmission electron microscope. However, using STXM, the morphological structures as well as the composition can be studied without any sample modification at high spatial resolution similar to an electron microscope. The embedding resins commonly used during sample preparation may sometimes interfere with the chemical mapping. Fig 8 shows the soft X-ray spectra of two commonly used resins for sample preparation. Both have spectral features in the region of interest of plant biopolymers. In comparison, LR white resin have spectral features very close to the amide peak at 288.2 eV, carboxyl group at 288.5, and the carbonate peak at 290.3 eV. Therefore, careful consideration should be given in choosing the resin depending on the biopolymer information required from the sample. Alternatively, cryo-ultramicrotome should be explored as an alternative to cut plant tissues without using an embedding medium.

The applications of soft X-ray spectromicroscopy for characterizing bio-composite samples, specifically the interface regions that are hundreds of nanometer in size have been shown through different studies [65,66]. The electron microscopes commonly used to characterize interfacial bonding in bio-composites cannot differentiate the interface regions from the fibre and polymer matrices. This limits the ability to characterize adhesion and the bonding at the adhesion regions in bio-composites. It is also difficult to determine if any polymer is impregnated into the fibres during bio-composite preparation, which may affect bio-composite quality. The carbon XAS measurements at high spatial resolution of 30 nm using the STXM are used to characterize the fibre, polymer matrix, and fibre-polymer matrix in flax bio-composite samples. The method has been even used to determine the composition of bean and quinoa chromosomes [40,41,67] and to visualize the effects of cell wall degrading enzymes on wood cells [68]. These studies clearly show the advantage of soft X-ray spectromicroscopy for biopolymer characterization without the need for labelling as it is required for other biopolymer characterization methods. Another advantage of the synchrotron based soft X-ray spectromicroscopy is the access to several elements, for example mapping organic and inorganic molecules for in-situ chemical analysis of plant samples.

Transmission and fluorescent modes are common methods of data collection in STXM. Elements or compounds of higher concentrations (a couple of percentage) can be easily detected by the transmission detector which takes much less time than the fluorescent mode [69]. The fluorescent detectors have high detection sensitivity and can detect a couple of ppm concentration compounds.

#### Relative Merits of Using Soft X-ray and FT-IR Spectromicroscopy Techniques

Sample preparation procedures for infrared and soft X-ray spectromicroscopy are similar. For both the techniques, the samples can be prepared using embedding medium or can be cryo-cut without using any embedding medium. The sample thickness requirement for both the techniques is different. However, the ultra-microtomes can cut alternate sections from the same sample for both the techniques. Samples mounted on Si_3_Ni_4_ can be also used for infrared spectromicroscopy. The samples can be kept dry or hydrated for soft X-ray whereas hydrated samples may be difficult for infrared spectromicroscopy.

Infrared has the advantage of less radiation damage compared to soft X-rays due to less intense beam and live cell samples can be kept alive even after data collection [8]. Soft X-rays are ionizing radiation and may induce damage to samples due to disassociation of sample chemical bonds or by deposition of organics in the X-ray beam path on the samples [41,70]. Radiation damage alters the spectral details of biopolymers and depends on the sample, sample state, rate of data collection, pixel resolution, number of repeated scans (or spectral resolution), and the beam characteristics. The radiation sensitivity of different biopolymers is different which should be taken into consideration when using STXM [39,41]. It has been shown that at same data collection rate and beam characteristics, cellulose from oak cell wall is prone to less damage compared to pure cellulose acetate. Wet bean chromosomes are damaged more than dry chromosomes and different fixatives used alter the rate of damage. The radiation damage can be checked by scanning the same region after data collection and at certain energies (286.7 eV) the extent of the damage is mostly visible. Further, increased intensities of aromatic and keto-enol regions at 285 and 286.6 eV of cellulose is a good indicator of radiation damage. The lentil stem samples in this study were scanned with the smallest dwell time possible (i.e. 1 ms) and number of repeated scans in the same region were limited by selecting optimal spatial (100 nm) and spectral (0.2 eV) resolutions, and limiting the energy scan range from 280–300 eV. The reference spectra samples were collected by defocussing the beam which significantly minimizes radiation damage. Careful handling of the sample such as selecting a fresh region after optimizing the STXM in an unwanted region in the sample dramatically helps to reduce the radiation damage from the region of interest. Sample cooling will reduce radiation damage and is possible in ultra-high vacuum (UHV) STXMs. A few UHV-STXMs are available in the world now and a few are being built. The similarity between soft X-ray and infrared methods enable one to combine the advantages of both the methods to study biopolymer samples and various synchrotrons around the world have both the techniques available in the same facility. An excellent review on sample preparation methods and requirements, instrumentation, and data analysis of synchrotron based of FT-IR and soft X-ray spectromicroscopy techniques for applications in environmental science is presented by Lawrence and Hitchcock [29].

The intrinsic challenge with plant samples is the complex molecular environments due to the presence of a large number of different biopolymers. The spectra of complex mixtures are usually dominated by the absorption of the polymer that is present in higher concentrations which may be problem in both soft X-rays and infrared spectromicroscopy. For instance, the soft X-ray spectra extracted from the cell walls did not show the strong pectin peak at 288.5 eV. Few possible explanations are: pectin in lentil stem is present in concentrations lower than the detection limit (<2%); pectin may have been removed during the sample preparation process; or the spectrum is dominated by presence of lignin, cellulose and hemicellulose in very high concentrations. The use of fluorescent probes and antibodies to identify specific proteins or sub-cellular components has been used in infrared spectromicroscopy in conjunction fluorescence microscopy. Similarly, the use of X-ray excited fluorescent probes to use with the high resolution STXM to overcome the limitations of molecular sensitivity in biological samples was explored but not much work has been pursued [60,71]. On the other hand the use of fluorescent probes to study the samples using confocal laser microscopy first and then using STXM has been demonstrated on river biofilm samples [30]. Similar approach can be very valuable for studying plant biopolymer samples using STXM. Further, if the sample feature is more than 100 nm or 8 um thick for the soft and IR data collection, respectively the spectra will have chemical information from other sample overlapping features. Three dimensional imaging is possible in both soft X-ray and IR spectromicroscopy and it will eliminate the spectral contamination from sample overlapping features [72,73].

## Conclusions

FT-IR spectroscopy is a powerful technique to differentiate all plant biopolymers. However, the limited spatial resolution made it difficult to study the distribution and association of biopolymers with different parts of the cell. The soft X-ray XAS has chemical speciation capabilities for most plant biopolymers and the carbon 1s spectra have good spectral features to differentiate biopolymers than the oxygen 1s spectra. The few peaks in soft X-ray spectra make the interpretation easier compared to IR spectroscopy. The sub-cellular spatial resolution that can be achieved using soft X-ray microscopy made it possible to quantitatively map the distribution of different biopolymers on plant cells. FT-IR spectromicroscopy is useful to characterize larger samples in a short time while soft X-ray spectromicroscopy is the good tool to provide speciation at high spatial resolution in smaller regions of interest. Both soft X-ray and FT-IR spectromicroscopy techniques are complementary to each other for in-situ characterization of biopolymers in plant samples.
